# New Treatment Opportunities in Phosphatase and Tensin Homolog (PTEN)-Deficient Tumors: Focus on PTEN/Focal Adhesion Kinase Pathway

**DOI:** 10.3389/fonc.2017.00170

**Published:** 2017-08-09

**Authors:** Roberta Alfieri, Elisa Giovannetti, Mara Bonelli, Andrea Cavazzoni

**Affiliations:** ^1^Department of Medicine and Surgery, University of Parma, Parma, Italy; ^2^Department of Medical Oncology, VU University Medical Center, Amsterdam, Netherlands; ^3^Cancer Pharmacology Laboratory, AIRC Start Up Unit, University of Pisa, Pisa, Italy

**Keywords:** phosphatase and tensin homolog, focal adhesion kinase, targeted therapy, kinase, drug combination

## Abstract

Deep genetic studies revealed that *phosphatase and tensin homolog* (*PTEN*) mutations or loss of expression are not early events in cancer development but characterize tumor progression and invasion. Loss of PTEN function causes a full activation of the prosurvival phosphoinositide 3-kinase (PI3K)/AKT/mTOR pathway, but the treatment with specific inhibitors of PI3K/AKT/mTOR did not produce the expected results. One of the alternative targets of PTEN is the focal adhesion kinase (FAK) kinase, mainly involved in the control of cancer cell spread. The connection between PTEN and FAK has been demonstrated in different tumor types, with reduced PTEN activity often correlated with increased expression and phosphorylation of FAK. FAK inhibition may thus represent a promising strategy, and some clinical trials are testing FAK inhibitors alone or combined with other agents in a number of solid tumors. However, only few preclinical and clinical data described the effects of the combination of PI3K/AKT/mTOR and FAK inhibitors. Increasing knowledge on the PTEN/FAK connection could confirm PTEN as a good prognostic marker for a combination strategy based on concomitant inhibition of PI3K/AKT and FAK signaling, in advanced metastatic malignancies with altered or reduced PTEN expression.

## Introduction

The development of new approaches directed to evaluate molecular alterations in cancer cells represents a key goal to better define the intrinsic characteristics of tumors and to explore molecular targets that are commonly deregulated in cancer cells. Novel insights regarding the second most commonly mutated tumor suppressor gene *phosphatase and tensin homolog* (*PTEN*) have been reported: loss of *PTEN* has been documented in a variety of solid tumors, including lung (1), prostate (2), colorectal (3), and brain (4) cancers. Typically, loss of function of PTEN causes aberrant activation of the phosphoinositide 3-kinase (PI3K)/AKT pathway, associated with increased malignant transformation and progression.

Recently, a series of new targets have been associated with PTEN phosphatase activity. PTEN is indeed a critical protein in the intracellular compartment, and controls, by its phosphatase activity, several important proteins, including signal transducers and activators of transcription (STAT) (5), Jun N-terminal kinase (JNK) (6), extracellular signal regulated kinase (ERK1/2) (7), AKT (8), and focal adhesion kinase (FAK) (9).

In this review, we will focus on the correlation between PTEN and one of its kinase targets, FAK.

## Regulation and Role of PTEN

*Phosphatase and tensin homolog* is located on chromosome 10q23 and encodes for a lipid phosphatase that converts PtdIns(3,4,5)P3 to PtdIns(4,5)P2 (10), by inhibiting 3-phosphoinositide-dependent kinase (PDK1) and consequently protein kinase B (AKT) activation. Moreover, PTEN exerts a protein phosphatase activity toward different protein substrates (5–9). PTEN is a member of the type-I protein tyrosine phosphatase family, composed by 403 amino acids organized in five functional domains: a PtdIns (4,5)P2 binding domain, a phosphatase domain, a C2 domain involved in targeting protein to cell membrane, a carboxyterminal region and a protein interaction (PDZ) domain (11). PTEN loss causes accumulation of PtdIns (3,4,5)P3, with recruitment and activation of AKT mediated by PDK1; PDK1 phosphorylates AKT at the critical residue Thr308 (12), whereas the Ser473 residue of AKT is a substrate of mammalian target of rapamycin 2 complex (mTORC2) (13).

Phosphatase and tensin homolog function can be compromised by different mechanisms including genetic alterations, transcriptional repression, microRNA regulation, promoter hypermethylation, and post-translational modifications.

Germ line mutations are present in 60–80% of patients with hamartoma tumor syndromes and Cowden disease (14). Somatic alterations, such as mutations, insertions and deletions have been found throughout the full gene and are not specific for a peculiar cancer type. PTEN inactivation is also a consequence of promoter hypermethylation (15) and reduction of transcription: Sal-like protein 4 and SNAIL can bind PTEN promoter mediating its repression through the interaction with the Mi-2/NuRD complex (16), and competition with p53 (17), respectively.

At post-transcriptional level, PTEN is downregulated by small non-coding RNAs. Some miRNAs (a class of 20–25 nucleotide non-coding RNA) that partially match with the 3′UTR region of PTEN mRNA are able to abrogate PTEN expression: in particular, mir-21, mir-22, mir-25A, and mir-200 family member (miR-200a and miR-200b) stably reduce PTEN protein levels (18–20). More recently, miR-93 has also been reported to reduce PTEN expression in non-small cell lung cancer (NSCLC) by directly targeting PTEN mRNA (21).

Finally, PTEN can be regulated at post-translational level. PTEN phosphorylation at Ser380, Ser385, Thr382, and Thr383 reduces its phosphatase activity by moving PTEN from the intracellular membrane level to the cytosol (22, 23). This event prevents its ability to interact with PtdIns(3,4,5)P3, which is located to the internal side of the plasma membrane. Reduction of PTEN activity is also observed after phosphorylation at residues Ser362 and Thr366 by glycogen synthase kinase 3-beta (GSK3-β), an enzyme activated by constitutive stimulation of the PI3K/AKT pathway (24).

Inactivation or loss of PTEN expression has been reported in different solid tumors including NSCLC, breast, colorectal, endometrial, and ovarian cancers (Table 1): in NSCLCs, altered PTEN expression, has been detected in 8.2–59% and in 2.1–46% of squamous cell lung cancer (SCC, squamous hystotype) and adenocarcinoma (AD) hystotype, respectively (25). Breast cancer tissues show significant reduction of PTEN expression compared to non-tumor tissues, and a meta-analysis (26) reported a positive correlation between PTEN loss and later TNM stage, evidencing that PTEN loss is not an early event, but it is associated with tumor progression. Patients with colorectal cancer display increasing inactivation of PTEN expression, as a consequence of promoter hypermethylation, decreased DNA copy number, and a general reduction of protein expression. All these alterations are associated with increased stage of disease; 20% of PTEN loss has been detected in stage I, while up to 58.9% has been found in stage IV (27). In prostate cancer, a high frequency of PTEN loss (between 16 and 41% of tumor samples) (2) has been described, being homozygous deletion the most frequent event. The clinical trials considering PTEN alteration among the inclusion criteria in different solid tumors are reported in Table 1.

**Table 1:** Clinical trials with PTEN alteration as inclusion criteria (http://clinicaltrials.gov/).

Agent	Target	In combination with	Tumors	Phase	Reference
GSK2636771	PI3K-β	–	AST	I/IIa	NCT01458067
		Paclitaxel	Gastric	I/II	NCT02615730
		Enzalutamide (AR inhibitor)	Prostate	I	NCT02215096
AZD8186		Abiraterone (CYP 17 inhibitor)	Prostate	I	NCT01884285
		AZD2014 (mTOR inhibitor)	SCC		
			Breast		
BKM-120	Pan-PI3K	Lapatinib (dual EGFR/HER2 inhibitor)	Breast	I/II	NCT01589861
		Carboplatin, paclitaxel	AST	I	(28)
		Abiraterone (CYP17 inhibitor), prednisone	Prostate	I	NCT01741753
		–	Thyroid	II	NCT01830504
		Paclitaxel	Breast	II/III	NCT01572727
		–	SCC	II	(29)
		–	Endometrial	II	NCT01550380
		RAD001	Lung	I	NCT01470209
MK2206	AKT	–	Ovarian, peritoneal	II	NCT01283035
		–	Breast	II	NCT01277757
		–	Colorectal	II	NCT01802320
GDC-0068		5-Fluorouracil, leucovorin, oxaliplatin	Gastric	II	NCT01896531
		Paclitaxel	Breast	II	NCT02162719
AZD5363		–	AST	I	NCT01226316
ARQ751	Pan-AKT PI3K/mTOR mTORC1	–	AST	I	NCT02761694
BEZ235		–	Perivascular epithelioid cell tumors	II	NCT01690871
RAD001		–	AST	II	NCT02449538
		Pazopanib (PAN-TKI)	AST	I	NCT01430572
		Paclitaxel	Breast	II	(30)
		5-Fluorouracil, epirubicin, cyclophosphamide (FEC)			
CCI-779		–	Prostate	I	NCT00235794
Erlotinib	EGFR	–	Glioblastoma, astrocytoma	I/II	(31)
Panitumumab	EGFR/HER-2	Carboplatin, gemcitabine	Breast	II	(32)
Trastuzumab	HER-2	RAD001	Breast	I/II	(33)
Sorafenib	PAN-TKI	RAD001	Thyroid	II	NCT01141309
INC-280	c-MET	BKM-120	Glioblastoma	II	NCT01870726
ASN003	B-Raf	–	AST	I	NCT02961283
GDC-0973	MEK	GDC-0068	AST	I	(34)
BMN 673	PARP	–	Endometrial	II	NCT02127151
MK-4827		–	Endometrial	II	NCT03016338

*PAN-TKI, multikinase inhibitor; AST, advanced solid tumor; SCC, squamous cell lung cancer*.

As a regulator of the PI3K-AKT-mTOR pathway, PTEN controls many intracellular processes promoting cancer growth, cell metabolism, angiogenesis and cell motility (25, 35). In particular, it regulates the plasma membrane expression of GLUT1 either by its lipidic phosphatase activity on PtdIns(3,4,5)P3 or by direct dephosphorylation of AKT (8). In addition, PTEN decreases the levels of pyruvate kinase isozyme M2 and 6-phosphofructo-1-kinase/fructose-2,6-biphosphatase isoform 3, both involved in the glycolytic process, thus exerting an anti-Warburg effect (36).

Loss of PTEN also influences cell polarization and motility, e.g., increased PtdIns (4,5)P2 level in normal cells attracts CDC42 to the apical membrane establishing cellular polarization after binding with PAR-6/aPKC complex (37). Loss of PTEN prevents the acquisition of this epithelial characteristic, increasing the possibility of cells to undergo epithelial to mesenchymal transition (EMT) (38). Similar to the effects obtained by complete loss of PTEN, two point inactivating mutations (C124S and G129E) cause acquisition of a highly malignant phenotype, whereas single allelic loss produces an intermediate effect (39).

Recently, the loss of PTEN has been described as a mechanism of resistance to immune checkpoint blockade in solid tumors. In melanoma models the loss of *PTEN* induces immunoresistance prompting VEGF and other immunosuppressive cytokines expression (40); similarly, *PTEN-*null prostate tumors suppress antitumor immune response by activating the JAK2-STAT3 pathway (41). The correlation between *PTEN*-loss and immunotherapy resistance has been also confirmed by evaluating PTEN expression in patients with metastatic melanoma treated with anti–PD-1 antibodies: PTEN expression is generally related to a greater reduction of tumor size in respect to PTEN-negative tumors (40). Accordingly, in a case report of uterine leiomyosarcoma, immunotherapy resistance is associated with biallelic *PTEN*-loss (42).

## Regulation and Role of FAK Expression

Focal adhesion kinase is a key regulator of the focal adhesion complex, which controls various intracellular processes such as cell motility (43), invasion (44) cell growth, and survival (45, 46), by regulating signals from integrin-mediated cell–extracellular matrix (ECM) connection (47) and from membrane receptors with kinase activity (48). Moreover the role of FAK as a regulator of tumor-infiltrating immunosuppressive cells (TILs) has been recently demonstrated in a mouse model of human pancreatic ductal AD (49). FAK maps on chromosomal region 8q24.3 and gene amplification has been discovered in gastric cancer (50), whereas increased mRNA levels have been detected in ovarian, head and neck and metastatic breast carcinoma (51). Currently, a part from some polymorphisms revealed by DNA sequencing, no mutational activation has been detected in this gene. As a kinase, it is composed by different domains: a FERM domain (protein band 4.1-ezrin-radixin-moesin homology domain), a central kinase domain, three prolin-rich regions, and the focal adhesion targeting domain (51).

After FAK homodimerization, as a consequence of clustering of integrin receptors by cellular adhesion to ECM, an autophosphorylation reaction occurs at Tyr397 residue (52). With the recruitment of the SRC-family kinases at Tyr397, FAK is then phosphorylated at the residue Tyr925 of the kinase domain. The FAK-SRC complex activates a plethora of different substrates, including paxillin, Shc, p120RAsGDP, and PLCy (53). The main targets of FAK, p130Cas and paxillin, are involved in migration, by modulating the expression and activation of members of Rho family GTPases; after FAK activation, p130Cas and paxillin promote focal adhesion complex formation, maturation, and turnover (54). FAK involvement in invasiveness might be at least in part explained by the fact that FAK promotes expression and activation of metalloprotease-9 (MMP9), as reported in breast (55) and lung (56) cancers. MMP9 mediates matrix degradation, causing tumor cell spreading. In agreement with the acquisition of a malignant phenotype, FAK activation also controls EMT process. As reported for breast cancer (57), the insulin growth factor receptor 1 (IGF1R)-driven migration and invasion are predominantly mediated by FAK activation; moreover, transforming growth factor β, a well-known cytokine involved in EMT induction, causes FAK activation, triggers delocalization of E-cadherin from the plasma membrane, and increases levels of mesenchymal markers (58). Interestingly, some evidences concerning the connection between FAK and β-catenin are emerging: in colorectal cancer, a high expression of FAK activates the Wnt/β-catenin signaling by phosphorylating GSKβ^Y216^ (59) with subsequent stabilization of β-catenin protein. Recent data enforced the rationale of targeting FAK in a cohort of lung AD patients with *KRAS* mutation, which is typically detectable in the percentage of 15–25% of NSCLC patients. KRAS promotes tumor growth, by constitutive activation of RhoA, which in turn activates FAK, causing the acquisition of a more aggressive phenotype that could be reverted by pharmacological inhibition of FAK kinase (60).

The lack of NF2 tumor suppressor gene, coding for Merlin protein, is a frequent event in mesothelioma. Merlin negatively regulates some targets including Src-FAK complex (61), and its deficiency could represent a potential biomarker for the treatment with FAK inhibitors: specific FAK inhibition of Merlin-negative mesothelioma cancer cells causes a relevant reduction in cell viability, suggesting the potential benefit of this approach in NF2 negative mesothelioma cancer patients (62). FAK targeting strategies in different solid tumors are reported in Table 2.

**Table 2:** Clinical trials involving specific focal adhesion kinase (FAK) inhibitors (http://clinicaltrials.gov/).

FAK inhibitor	In combination with	Tumors	Phase	Reference
VS-6063	–	Mesothelioma	II	NCT01870609
	–	Mesothelioma	II	NCT02004028
	–	NSCLC, KRAS mutant	II	NCT01951690
	–	AST	I	(63)
	–	AST	I	(64)
	Pembrolizumab (anti PD-1)	NSCLC, mesothelioma, pancreatic cancer	I/IIA	NCT02758587
	Pembrolizumab, gemcitabine	AST	I	NCT02546531
	Paclitaxel	Ovarian	I	NCT01778803
	Avelumab (anti-PD-L1)	Ovarian	I	NCT02943317
	VS-5584 (dual PI3K/mTOR inhib)	Mesothelioma	I	NCT02372227
GSK2256098	Trametinib (MEK inhibitor)	Mesothelioma, AST	I	NCT01938443
	–	AST	I	NCT01138033
VS-4718	Gemcitabine, Nab-paclitaxel	Pancreatic cancer	I	NCT02651727
PF00562271	–	AST	II	(65)

Several evidences demonstrated that FAK could be directly activated by PI3K/AKT pathway. In particular, the AKT-1 isoform is implicated in FAK activation, by directly phosphorylating three critical Serine residues (Ser517/601/695). These events promote FAK^Y397^ autophosphorylation and thus enzyme activation. Notably, the cooperation between FAK and AKT is bidirectional: depletion of FAK reduces AKT^S473^ phosphorylation, evincing that a mutual cooperation could be responsible for cancer progression, at least in certain tumor types (66). Interestingly, novel data provided further insights on FAK and AKT correlation (67): in a preclinical model of ovarian cancer, the protein tyrosine phosphatase non-receptor type 12 (PTPN12) dephosphorylates FAK, shutting down the migratory properties. PTPN12 is downregulated after Her-2 activation, with reduction of PTEN protein and constitutive activation of PI3K/AKT pathway. In particular, AKT phosphorylates and inactivates GSK3 protein that in turn dephosphorylates FAK at the inhibitory target Ser722, promoting cell spread.

## Connection Between PTEN and FAK Signaling Pathways

The connection between PTEN and FAK has been explored for few years but only recently this has emerged as a potential druggable signaling axis.

The interaction between PTEN and FAK is clearly demonstrated in MKN28 cells, a preclinical model of gastric cancer (68) which shows a significant reduction of FAK expression and activation when overexpressing PTEN, with inhibition of cell motility, invasiveness and *in vivo* tumor growth.

A negative correlation between PTEN and FAK is also detected in patients with multiple myeloma (MM) (69) and urologic malignancies (70) in advanced stage. Moreover, it has been reported that Notch1 controls PTEN expression in hepatocellular carcinoma: the abrogation of Notch1 by siRNA approach increases PTEN expression and phosphorylation, with inhibition of both AKT and FAK activity (71).

Loss of PTEN function, a frequent event reported in endometrial cancer (72), with percentage near to 60%, makes this mutation an attractive molecular target in this type of cancer. In a preclinical study, cell lines with wild-type or mutated *PTEN* show different sensitivity to the specific FAK inhibitor GSK2256028 given alone or combined with chemotherapy. In an *in vivo* model, the combined treatment shows a dramatic effect only in cells carrying mutated *PTEN*, with apoptosis induction, reduced cell growth and neo-angiogenesis. To further sustain this correlation, a cohort of 91 patients was analyzed for PTEN and FAK expression and phosphorylation at Tyr397. Patients with poor prognosis show reduced PTEN levels associated with increased FAK expression and Tyr397 phosphorylation, confirming that PTEN could be considered a prognostic biomarker and suggesting its role for predicting the response to anti-FAK targeted agents (73).

*Phosphatase and tensin homolog* mutations are detected in 15–25% of patients with acute lymphoblastic leukemia (T-ALL). However, while *in vitro* experiments demonstrated that the pharmacological inhibition of PI3K/AKT/mTOR pathway in PTEN null-T-ALL cells dramatically reduces cell growth and viability, the efficacy of this treatment is less pronounced in an *in vivo* system. As previously reported for MM, gastric and endometrial cancers, *PTEN* null-T-ALL cells display increased FAK activity. Genetic deletion or pharmacological inhibition of FAK, associated with inhibition of PI3K pathway, produce a more significant effect than single monotherapy (74) in this cellular model.

In our recent article (75), we analyzed the correlation between PTEN loss and FAK activation in squamous NSCLC patients. As previously reported (76), PTEN levels are reduced in 70 and 77% of patients with lung squamous (SCC) or AD histology, respectively. This event, observed in metastatic patients, has been confirmed by our analysis in a cohort of 51 patients with SCC, at different stage (I–IV). In particular, we demonstrated that loss of PTEN expression is not an early event, at least in SCC, but it is associated with cancer progression and acquisition of high malignancy: in fact, the majority of metastatic stage IV patients presents low PTEN expression associated with increased FAK^Y397^ phosphorylation. Cell clones, expressing low level of PTEN, demonstrated increased FAK^Y397^ and AKT^S473^ phosphorylation, high miR-21 levels, and the acquisition of a mesenchymal phenotype.

The acquisition of mesenchymal markers caused by PTEN abrogation can be a consequence of increased onco-miR levels: in particular, the miR-130 family, highly expressed in bladder cancer, shuts down PTEN protein, with increase of migration and invasiveness, MMP9 production, AKT, and FAK phosphorylation (77). Similarly, miR-301a is upregulated in tumor tissue from melanoma patients (78), and modulation of this miRNA in cancer cells revealed lower PTEN expression, with activation of both AKT and FAK.

Notwithstanding this connection is reported for different tumor types, it cannot be extended to all cancers: for example, esophageal squamous cell carcinoma cells depleted for PTEN (79) show increased malignancy, but the mechanism of metastatic spread was not correlated with FAK activation.

Finally, in most studies the connection between PTEN and FAK is based on the phosphatase activity of PTEN, but recent data (80) demonstrated that FAK could in turn phosphorylate PTEN^Y336^, increasing protein stability and phosphatase activity. This result shows a novel mechanism of PTEN regulation providing new insights for the role of FAK.

## Targeting PTEN/FAK Signaling

Taking into account the relevance of genetic *PTEN* aberrations such as loss or reduced expression, point mutations or post-translational events, and the critical role of PTEN enzyme as lipid and protein phosphatase, it is of particular interest to approach new targeted strategies based on PTEN status evaluation (Table 1). PTEN, as tumor suppressor gene, cannot be considered a classical oncogenic driver such as EGFR or EML4/ALK in NSCLC patients or BRAF in melanoma patients, but it can constitutively regulate some intracellular oncogenic signaling pathways, such as the PI3K/AKT/mTOR axis. A high number of specific or pan inhibitors of this pathway have been developed, and novel molecules are continuously synthesized, but to date, no specific therapies are approved for patients with PTEN deficiency. This could be a consequence of the pleiotropic effects exerted by PTEN depletion; as reported, abrogation of this protein causes not only activation of PI3K/AKT pathway, but increases JNK, MAPK, STAT, and FAK activity, making problematic targeting this multiple molecular alterations. Moreover, PTEN alterations are often acquired during the last phase of tumor progression; in particular loss of PTEN can be associated with the development of metastatic disease and it can be considered a marker of advanced tumor stage.

Another important aspect emerging from preclinical studies is the pivotal role of PI3K-β as an enzyme often overexpressed in the presence of PTEN loss (2, 81). This enzyme can be targeted by specific (GSK-2636771, AZD8186 or TGX-221), pan (GDC-0032, XL-147, GDC-0941, NVP-BKM120, PX-866, BAY 80-6946), or dual PI3K/mTOR (NVP-BEZ235, XL-765) inhibitors. In a preclinical *in vivo* model characterized by PTEN loss, the selective inhibition of the β subunit of PI3K caused tumor shrinkage (82). Recently, a correlation between PTEN loss and high PI3K-β was reported in a cohort of NSCLC patients, with prevalence of squamous histology (83). Currently, four clinical trials (Table 1) (NCT01458067, NCT02215096, NCT02615730, and NCT01884285) are evaluating the role of specific PI3K-β inhibitors in patients with altered PTEN expression.

In view of the fact that PTEN is one of the most important regulators of FAK kinase, loss of PTEN can be regarded as a good biomarker for a combined therapy with PI3K and FAK inhibitors. Currently, this strategy (74) provides a better treatment for PTEN-null T-ALL cells compared to the inhibition of PI3K/AKT alone. The same approach has been tested by our group in a preclinical model on SCC with stable PTEN abrogation, demonstrating that a simultaneous inhibition of both targets induces synergistic reduction of cell growth and invasiveness (75).

This new combination deserves further investigations in the clinical practice. However, to date there is only a phase I clinical trial (NCT02372227) exploring the safety of the PI3K/AKT/mTOR inhibitor VS-5584 combined with the FAK inhibitor VS-6063 in patients with relapsed malignant mesothelioma (Table 2). Remarkably, since PTEN loss is typically both in advanced primary tumors and in metastatic sites, the development of a combined strategy directed to inhibiting PI3K aberrant activation caused by PTEN loss and FAK kinase could represent a promising strategy to target metastases, which are the leading cause of death in cancer patients.

## Author Contributions

RA wrote the manuscript. EG revised the manuscript. MB performed bibliographic research. AC performed bibliographic research, planned, and wrote the manuscript.

## Conflict of Interest Statement

The authors declare that the research was conducted in the absence of any commercial or financial relationships that could be construed as a potential conflict of interest.
